# Significance of tumor markers combined with neutrophil to lymphocyte ratio, D-dimer and T-lymphocyte in the diagnosis of colon cancer

**DOI:** 10.12669/pjms.39.4.7157

**Published:** 2023

**Authors:** Qianying Li, Shumei Geng, Xiaowei Zhang, Zilong Jia

**Affiliations:** 1 Qianying Li Department of Medical Oncology, Baoding No.1 Central Hospital, Baoding 071000, Hebei, China; 2 Shumei Geng Department of Medical Oncology, Baoding No.1 Central Hospital, Baoding 071000, Hebei, China; 3 Xiaowei Zhang Department of Medical Oncology, Baoding No.1 Central Hospital, Baoding 071000, Hebei, China; 4 Zilong Jia, Department of Ultrasonography Laboratory. Baoding No.1 Central Hospital, Baoding 071000, Hebei, China

**Keywords:** Colon cancer, Tumor markers, Neutrophil to lymphocyte ratio, D-dimer, T-lymphocyte, Diagnosis

## Abstract

**Objective:**

To analyze the value of combined detection of tumor markers, neutrophil to lymphocyte ratio (NLR), D-dimer and T lymphocyte in the diagnosis of colon cancer.

**Methods:**

This is a retrospective study. A total of 80 patients with colon cancer and 80 patients with benign colon mass admitted to Baoding NO.1 Central Hospital from June 10, 2021 to December 10, 2022 were divided into the study group and the control group. Further comparison was performed on the tumor markers, NLR, D-dimer and T-lymphocyte levels between the two groups, associated with the comparison of corresponding levels of colon cancer at different stages. In addition, correlation analysis was carried out focusing on the above indicators with colon cancer.

**Results:**

Carcinoembryonic antigen (CEA), CA199, NLR, D-dimer and CD8^+^ cell count levels in the study group were significantly higher than those in the control group, while CD4^+^ cell count and CD4^+^/CD8^+^ ratio were obviously lower (P<0.05). Among I-IV colon cancer, the highest levels of CEA, CA199, NLR, D-dimer, CD4^+^ and CD4^+^/CD8^+^ ratio were found in patients with Stage-IV colon cancer, while the level of CD8^+^ was the lowest (P<0.05). Correlation analysis indicated that CEA, CA199, NLR, D-dimer and CD8^+^ were positively correlated with whether the patient had colon cancer (r=0.841, 747,991,889,565, all P<0.05), but negative correlations with CD4^+^ and CD4^+^/CD8^+^ ratio (r=-0.999, -0.994, all P<0.05).

**Conclusion:**

The detection of tumor markers combined with NLR, D-dimer and T-lymphocytes has reference value in the diagnosis of colon cancer.

## INTRODUCTION

Colon cancer is a common malignant tumor of the digestive system in clinical practice, which occurs frequently at the junction of rectum and sigmoid colon.^1^-^3^ There is an absence of obvious symptoms or lack of specific changes in the early stage of colon cancer, which increases the difficulty of early diagnosis clinically. Most patients are already in the advanced stages of the disease at the time of diagnosis, which seriously threatens the quality of life as well as physical and mental health of patients.^4^ At present, it has been recognized that early diagnosis and treatment are crucial to prolong the survival time and improve the quality of life of colon cancer patients.^5^ Biopsy through colonoscopy biopsy can observe the lesion directly under the microscope and acquire biopsy tissues for pathological examination, which has been considered to be the “gold standard” for the diagnosis of colon cancer. 6 However, biopsy through colonoscopy is an invasive examination, patients in the early stage are not willing to receive colonoscopy when there is no obvious discomfort.^7^

Therefore, looking for better solutions and indicators for diagnosis to achieve early diagnosis and detection has become a key issue in the treatment of colon cancer. With the development of testing technology, increasingly more blood marker tests have been applied to the diagnosis of colon cancer in view of their advantages of non-invasive, simple and rapid detection. Among them, the most-used indicators are tumor markers, neutrophil to lymphocyte ratio (NLR), D-dimer and T-lymphocyte subsets. However, detection of the above indicators are all non-specific tests, with relatively low sensitivity and specificity when testing alone for diagnosing colon cancer.^8^

Accordingly, in order to further explore the diagnostic significance of tumor markers, NLR, D-dimer and T-lymphocyte subsets in patients with early colon cancer, we retrospectively analyzed and compared the clinical data of 80 patients with colon cancer and 80 patients with benign colon mass and to explore the application value of tumor markers, NLR, D-dimer and T-lymphocyte subsets in early diagnosis of colon cancer, so as to provide reference for clinical work.

## METHODS

This is a retrospective study. A total of 80 patients with colon cancer and 80 patients with benign colon mass admitted to Baoding NO.1 Central Hospital from June 10, 2021 to December 10,2022 were divided into two group: the study group (colon cancer) and the control group (benign colon mass). The study group including 55 males and 25 females, with an average of 55.43±6.93 years old (43-65 years). Meanwhile, the control group including 53 males and 27 females, with an average of 57.35±5.97 years old (48-66 years). The enrolled 80 patients with colon cancer were divided according to the Dukes staging, including 22 patients with Stage-I, 30 patients with Stage-II, 15 patients with Stage-III and 13 patients with Stage-□.

### Ethical Approval:

The study was approved by the Institutional Ethics Committee of Affiliated Hospital of Baoding NO.1 Central Hospital, and written informed consent was obtained from all participants (No: [2022]060; Date: November 03, 2022).

### Inclusion criteria:

- patients confirmed by pathological examination;
- patients without mental illness and cognitive impairment who could cooperate to complete the research;
- patients with complete clinical data; and
- patients who signed the consent form by themselves and their families and were able to cooperate in completing the study.

### Exclusion criteria:

- patients with other malignant tumors;
- patients with both autoimmune and hematological diseases;
- patients with acute and chronic infection;
- patients with severe liver, kidney, brain and other important organ dysfunction;
- patients with intestinal obstruction; and
- patients with mental illness or those who cannot cooperate with the study for other reasons.

There was no statistical difference between the two groups in terms of general data, suggesting the comparability between the two groups (Table-I). Blood samples were collected in all cases under fasting condition in the morning. Serum carcinoembryonic antigen (CEA) and carbohydrate antigen 199 (CA199) were determined by enzyme-linked immunosorbent assay (ELISA). The neutrophil and lymphocyte counts of peripheral blood were detected by nucleic acid fluorescence staining using full-automatic blood cell analyzer to calculate NLR. The content of plasma D-dimer was determined by immunoturbidimetry. Besides, T-lymphocyte subsets in peripheral blood were determined by flow cytometry. A comparative analysis was performed on the levels of CEA, CA199, NLR, D-dimer as well as CD4^+^, CD8^+^ and CD4^+^/CD8^+^ in the two groups.

**Table-I:** Comparison of general data between the two groups (*χ̅*±*S*) n=80.

Indicators	Study group	Control group	t/χ^2^	p-Value
Male (n,%)	55 (68.75)	53 (66.25)	0.114	0.736
Age (years)	55.43±6.93	57.35±5.97	1.883	0.061
Hypertension (n,%)	11 (13.75)	14 (17.50)	0.427	0.514
Diabetes (n,%)	13 (16.25)	12 (15.00)	0.047	0.828
Obesity (n,%)	12 (15.00)	16 (20.00)	0.693	0.405
Bad diet habits (n,%)	22 (27.50)	18 (22.50)	0.533	0.465

p>0.05.

### Statistical analysis:

SPSS 20.0 software was used for statistical analysis. According to the normality analysis of measurement data, normally distributed data were expressed as (*χ̅*±*S*) and analyzed using two independent sample t-tests; while non-normally distributed data were expressed as median (quartile), Mann-Whitney U-test was used for two independent sample test, and Kruskal-Wallis H-test for comparative analysis of multiple independent samples. Counting data were showed in the form of absolute value or composition ratio, and compared using χ^2^ test. P<0.05 was used to indicate the presence of statistically significant difference.

## RESULTS

CEA, CA199, NLR, D-dimer and CD8^+^ cell count levels in the study group were significantly higher than those in the control group (P<0.05); compared with the control group, the study group had lower CD4^+^ cell count and CD4^+^/CD8^+^ ratio but higher CD8^+^ cell count, with statistically significant differences (P<0.05), as shown in Table-II.

**Table-II:** Comparison of CEA, CA199, NLR, D-dimer and T-lymphocyte subsets levels between the two groups (*χ̅*±*S*) n=80.

Indicators	Study group	Control group	t/Z	p-Value
CEA (ng/ml)	8.70(5.73,10.98)	1.00(0.70,1.60)	10.872	0.000
CA199 (ng/ml)	13.10(12.225,15.80)	9.20(7.80,9.90)	9.692	0.000
NLR	2.80(2.64,2.88)	1.91(1.35,3.12)	7.943	0.000
D-dimer (ng/ml)	326.28±12.98	127.19±13.71	94.337	0.000
CD4+(%)	325.20(323.98,327.08)	525.90(524.45,527.38)	10.922	0.000
CD8+(%)	498.40(486.13,501.28)	454.65(443.38,457.40)	10.880	0.000
CD4+/CD8+	0.65(0.65,0.67)	1.16(1.15,1.20)	10.920	0.000

Further comparison revealed that the levels of CEA, CA199, NLR and D-dimer were incomplete identical in peripheral blood of patients with I-IV colon cancer. Among them, the highest levels of CEA, CA199, NLR and D-dimer were found in patients with Stage-IV colon cancer (P<0.05; Table-III).

**Table-III:** Comparison of CEA, CA199, NLR and D-dimer levels in patients with colon cancer at different clinical stages.

Stages	CEA (ng/ml)	CA199 (ng/ml)	NLR	D-dimer (ng/ml)
I	5.30(4.23,5.50)*	11.75(10.50,12.10)*	2.82 (2.73,3.25)*	311.75 (309.68,313.70)*
II	8.40(7.35,9.30)*	12.95(12.50,14.10)*	2.85(2.83,2.90)*	324.75 (319.58,327.55)*
III	10.90(10.70,11.40)*	15.80(15.40,16.10)*	2.68(2.60,2.69)*	337.70(332.70,338.20)*
IV	13.50(12.40,14.60)*	17.80(16.95,18.70)*	2.50(2.25,2.58)*	343.70(341.20,343.70)*

******Note:******

* Kruskal-Wallis H test, P<0.05.

In Table-IV, it was detected that the levels of CD4^+^, CD8^+^ and CD4^+^/CD8^+^ ratio were incomplete identical in peripheral blood of patients with □-□ colon cancer. Among them, the highest levels of CD4^+^ and CD4^+^/CD8^+^ ratio and the lowest level of CD8^+^ were found in patients with Stage-IV colon cancer(P<0.05).

**Table-IV:** Comparison of T lymphocyte subsets in patients with colon cancer at different clinical stages.

Stages	CD4+(%)	CD8+(%)	CD4+/CD8+
I	320.30(318.40,323.45)*	503.80(501.85,509.75)*	0.64 (0.62,0.64)*
II	324.85(324.65,325.65)*	499.25(497.65,500.40)*	0.65(0.65,0.65)*
III	326.70(326.30,327.80)*	487.10(482.40,491.10)*	0.67(0.66,0.68*
IV	334.40(332.90,335.80)*	474.80(470.30,476.05)*	0.70(0.70,0.71)*

******Note:******

* Kruskal-Wallis H test, P<0.05.

Correlation analysis indicated that CEA, CA199, NLR, D-dimer and CD8^+^ were positively correlated with whether the patient had colon cancer (r=0.841, 747, 991, 889, 565, all P<0.05), but negative correlations were observed with CD4^+^ and CD4^+^/CD8^+^ ratio (r=-0.999, -0.994, all P<0.05).

## DISCUSSION

In recent decades, there is an increase in the incidence of colon cancer with the changes of people’s lifestyle and dietary structure.^9^,^10^ Colon cancer patients have no specific clinical manifestations in the early stage of onset, which increases the difficulty of clinical diagnosis. Some patients are in the advanced stages at the time of diagnosis, leading to the lost of the best opportunity for radical surgery and seriously affecting the prognosis of the diseased patients.^11^ According to previous research,^12^ about 20%~25% of the patients were in the advanced stages of the disease, approximately 1/3 of which might have recurrence or metastasis despite undergoing radical surgery, resulting in a poor prognosis.^13^ Therefore, the application of reliable detection technology to improve the early diagnosis of colon cancer is of great significance to strengthen the therapeutic effect and prolong the survival time of patients. Our study shows that the combined detection of tumor markers, NLR, D-dimer and T lymphocytes has reference value in the diagnosis of colon cancer.

Serum tumor markers are a class of chemicals synthesized and released by tumor cells that can react to cancer hosts.^14^ Recently, there is a continuous development of molecular biology technology, promoting an extensive application of tumor marker detection clinically, which plays an important role in determining the diagnosis, recurrence and prognosis of malignant tumors.^15^ Multiple serum tumor markers have been adopted clinically for the diagnosis of colon cancer at present, among which CEA and CA199 are the most common tumor markers in the diagnosis and treatment of colon cancer.

CEA is a broad-spectrum tumor marker which exists in colorectal, liver, pancreas and gastric tumor cells.^16^ CEA can be formed in normal human digestive gland cells but is detected with a relatively low serum content. It will affect the secretion of cells and can be highly expressed in blood after cell canceration.^17^ CA199 is a mucinous macromolecular glycoprotein, which is a diagnostic indicator for various malignant tumors, such as pancreatic and gastrointestinal malignant tumors. In the presence of malignant lesions, relevant antigens can be discharged from the blood circulation through the thoracic duct, exhibiting the elevation of CA199 in the blood.^18^ In our study, CEA and CA199 in patients with colon cancer were significantly higher than those in patients with benign lesions, with statistically significant differences (p=0.000). Moreover, there were also differences in the levels of CEA and CA199 in colon cancer patients at different stages. These levels were increased in the early stage than in the normal range, and were significantly higher in the advanced stages of the disease. In terms of the possible cause, we believe that it is related to the fact that most tumors have metastasis in the advanced stages, which may lead to the increase of CEA and CA199 reflex secretion. According to Attallah et al^19^, Serum levels of CEA and CA199 were increased in the order colon cancer > benign disease > healthy controls, this is similar to our research results.

Most patients with malignant tumors frequently have tumor-related hypercoagulable states, which can further promote the development of tumors.^20^ There may be high expression of plasminokinase, secretion of cross-linked fibrin, which may interact with coagulation factor 12 and thrombin, eventually leading to reflex elevation of the level of D-dimer. Consequently, the detection of D-dimer can help to determine the condition of malignant tumor and evaluate the prognosis of patients.^21^ In the present study, the level of D-dimer in colon cancer patients was significantly higher than that in the control group, and a higher level was observed in patients with higher tumor stage. This is consistent with the research of Kilic et al.^22^ It is speculated that in the early stage of colon cancer, the tumor tissue is relatively locally distributed, with mild damage to the balance of coagulation-fibrinolysis system; while in the advanced stages, there may already be local infiltration and distant metastasis in most cases, which may induce a serious damage to the balance of coagulation-fibrinolysis system, leading to a significantly increased level of D-dimer.

It is well known that various inflammatory cells (e.g., neutrophils and lymphocytes) infiltrate tumor cells in the tumor microenvironment. Inflammatory cells interact with tumors by secreting cytokines, which have an impact on the occurrence and development of tumors.^23^,^24^ It has been reported that neutrophils participate in enhancing the invasion and metastasis of tumor cells, while inhibiting anti-tumor immune surveillance; and many subpopulations of lymphocytes play a tumor role in tumor immunity.^25^ NLR, an inflammatory index used to predict the progression of cancer, is increasingly used in tumor diagnosis and treatment.^26^ Meanwhile, peripheral blood lymphocyte count can accurately reflect the immune status in vivo, which is of great significance for the early detection of patients and the monitoring of postoperative prognosis.^27^ In this study, the NLR of patients with colon cancer was significantly higher than that of the controls, and it was higher in the early stage of tumor than that in the advanced stages. Considering the potential cause, the tumor is locally distributed in the early stage, with relatively heavier local inflammatory reaction and immune reaction; while in the advanced stages, the tumor metastasizes frequently, resulting in systemic inflammatory reaction, and serious damage of the tumor to the immune system in vivo, which is reflected in the decrease of NLR. Simultaneously, the count of CD4^+^ cells and the ratio of CD4^+^/CD8^+^ in lymphocyte subsets of colon cancer patients were lower than that of the control group, while the count of CD8^+^ cells was higher than that of the control group. The major reason is that in the early stage of malignant tumors, there is a small change of systemic immune function in vivo, mainly local responses; however, the systemic immune function is gradually suppressed with the progress of tumors.

### Limitations:

It includes a smaller sample size without follow-up data, which restricted the integration of the research content with the final outcome of patients. In our future research, with more sample size and follow-up, it is expected to evaluate the clinical application value more objectively and accurately by combining the changes in the levels of relevant analyses with the outcome of patients.

## CONCLUSION

In addition to endoscopy and imaging, combined detection of tumor markers, NLR, D-dimer and T-lymphocyte subsets may contribute to early diagnosis of colon cancer, which may provide important reference for clinical diagnosis and treatment.

### Authors’ Contributions:

**QL** and **SG:** Carried out the studies, participated in collecting data, drafted the manuscript, are responsible and accountable for the accuracy and integrity of the work. **XZ:** Performed the statistical analysis and participated in its design.

**ZJ:** Participated in acquisition, analysis, or interpretation of data and draft of the manuscript. All authors have read and approved the final manuscript.
